# Body Localization of ACE-2: On the Trail of the Keyhole of SARS-CoV-2

**DOI:** 10.3389/fmed.2020.594495

**Published:** 2020-12-03

**Authors:** Francesca Salamanna, Melania Maglio, Maria Paola Landini, Milena Fini

**Affiliations:** ^1^Surgical Sciences and Technologies, Istituto di Ricovero e Cura a Carattere Scientifico Istituto Ortopedico Rizzoli, Bologna, Italy; ^2^Scientific Direction, Istituto di Ricovero e Cura a Carattere Scientifico Istituto Ortopedico Rizzoli, Bologna, Italy

**Keywords:** SARS-CoV-2, COVID-19, ACE2, ACE2 receptor, body localization

## Abstract

The explosion of the new coronavirus (SARS-CoV-2) pandemic has brought the role of the angiotensin converting enzyme 2 (ACE2) back into the scientific limelight. Since SARS-CoV-2 must bind the ACE2 for entering the host cells in humans, its expression and body localization are critical to track the potential target organ of this infection and to outline disease progression and clinical outcomes. Here, we mapped the physiological body distribution, expression, and activities of ACE2 and discussed its potential correlations and mutal interactions with the disparate symptoms present in SARS-CoV-2 patients at the level of different organs. We highlighted that despite during SARS-CoV-2 infection ACE2-expressing organs may become direct targets, leading to severe pathological manifestations, and subsequent multiple organ failures, the exact mechanism and the potential interactions through which ACE2 acts in these organs is still heavily debated. Further scientific efforts, also considering a personalized approach aimed to consider specific patient differences in the mutual interactions ACE2-SARS-CoV-2 and the long-term health effects associated with COVID-19 are currently mandatory.

## Introduction

### SARS-CoV-2 Clinical Characteristics

Since its discovery in December 2019 the coronavirus disease (COVID-19), caused by the transmission of a novel coronavirus known as SARS-CoV-2 induced pneumonia, infected more than 37,800,000 people worldwide and caused more than 1,080,000 deaths until October, 2020. COVID-19 patients mainly displayed pneumonia-associated symptoms, such as fever, shortness of breath, cough, sputum production, and myalgia or fatigue (1, 2). However, despite SARS-CoV-2 infection is manifested as a respiratory tract infection, it may causes symptoms associated to multiple organs, including intestine and stomach (diarrhea, anorexia, nausea, vomiting, and abdominal pain), liver (abnormal enzymes levels), pancreas (pancreatitis), kidney (protein and blood in their urine, abnormal creatinine level), brain (strokes, seizures, confusion, and brain inflammation), heart and blood vessels (elevations of cardiac injury biomarkers, palmus, chest distress, cardiac inflammation and injury, arrhythmias, and blood clots), eyes (conjunctivitis, inflammation of the membrane that lines the front of the eye, and inner eyelid), nose (anosmia), ect (3–11). This multiple organ involvement can lead to a poorer outcome to the viral infection and often result in hospitalization and intensive care unit (ICU) admittance (12–14). Despite the mechanisms for high morbidity and mortality induced by SARS-CoV-2 are currently unknown, based on available literature data in public databases, it is known that the risk of infection and mortality increases with advancing age and also seems to show a sexual dimorphism, male elderly subjects are at higher risk of infection, as well as death (1, 2). In addition, despite COVID-19 is a non-discriminatory disease, involving both healthy individuals and those with comorbidity conditions, it is well-documented that mortality further increases in presence of pre-existent pathologies, such as cardiovascular disease, hypertension, diabetes, obesity, chronic pulmonary disease, and cancer (12–14). Despite, the biological mechanisms behind these observations are still unclear, virus/host cell interaction, immunological differences, and sex-based hormonal differences are likely to be involved.

### Interaction Between SARS-CoV-2 and ACE2

Mechanisms implicated in SARS-CoV-2/host cell interaction are of key importance for cell infection and replication that in turn lead to disease and related damage. In this context, the angiotensin converting enzyme 2 (ACE2), an enzyme important in the renin-angiotensin-aldosterone system (RAAS), scarcely present in the circulation, but widely expressed in organs and able to regulate blood pressure and fluid balance, has been seen to play a key role (15, 16). ACE2 operates as an ACE counterpart: it acts as a carboxypeptidase, removing single amino acids, converting Ang II to its metabolite angiotensin-(1–7) (Ang1–7), balancing the effects of Ang II. ACE2 is found to the apical surface of epithelial cells, differently from ACE, which is located between the apical and basolateral membranes in polarized cells. ACE2 plays its pivotal role in regulating blood pressure and consequently hypertension. This activity is mediated by the ACE2/Ang-(1–7)/Mas receptor axis, through which the regulation of angiotensin and Ang-(1–7) and nitric oxide (NO) availability control blood pressure alterations, which cause damages to vascular tissue as atherosclerosis, hypertrophy and more in general, endothelial alterations (17). Operatively, there are two forms of ACE2: (1) the full-length ACE2, that presents a structural transmembrane domain able to anchor its extracellular domain to the plasma membrane, and (2) the soluble form of ACE2, that lacks the membrane anchor and circulates in small amounts in the blood (15, 16). SARS-CoV-2 enters cell by the binding of spike (S) viral protein, an amino acid long protein that belongs to the viral envelope and leans outwards with a “corona” like form, to the ACE2 receptor (16, 18). The initial step of viral entry is represented by the binding of the N-terminal domain of the viral protein unit S1 to a pocket of the ACE2 receptor. After this, the receptor transmembrane protease serine 2 (TMPRSS2), a member of the Hepsin/TMPRSS subfamily that is stechiometrically contiguous to ACE2 receptor, induces the cleavage of the protein between the S1 and S2 units, with the help of Furin which facilitates the entry of the virus into the cell after binding (19, 20). Furin [also termed paired basic amino acid cleaving enzyme (PACE)], a member of the subtilisin-like proprotein convertase family that processes protein of the secretory pathway, is expressed in multiple organs, such as in lungs, liver, and small intestines. Following the binding of the S glycoprotein to ACE2, furin-mediated proteolytic cut of the S protein is necessary for viral entry into the cell (18, 21). Thus, both TMPRSS2 and Furin are crucial for S activation. The key role of these two proteases was also demonstrated by a recent study that showed that multicycle replication of SARS-CoV-2 in Calu-3 human airway cells was strongly suppressed by inhibiting TMPRSS2 and Furin activity (22). However, virtually, other human proteases, e.g., cathepsin L and B, elastase, trypsin and factor X, may be involved in the entry of SARS-CoV-2 into the human cell and in the shedding of ACE2. A critical cell membrane protease involved in the endogenous shedding of ACE2 from membranes is the disintegrin metalloproteinase 17 (ADAM17), also known as tumor necrosis factor-α converting enzyme (TACE) (23). While TMPRSS2 cleaves both ACE2 and the S protein of SARS-CoV-2, leading to membrane fusion and cellular uptake of the virus, ADAM17 acts directly and solely on ACE2 and leads to ACE2 shedding into the extracellular cellular space. Thus, ADAM17 and TMPRSS2 may have opposite effects on ACE2 shedding. Evidences have shown that the expression of TMPRSS2 inhibits ADAM17-shedding of ACE2 (24). However, it is unclear how TMPRSS2 transcends ADAM17 to cleave ACE2 during SARS-CoV-2 infection.

Despite numerous information has been obtained up to now, the exact role of ACE2 in SARS-CoV-2 cellular infection and of proteases that process SARS-CoV-2 S protein is not yet defined. Certainly, genetics and demographic characteristics, lifestyle, comorbidities, and medication usage may have an impact on ACE2 expression and activity in SARS-CoV-2 cellular infection.

### Risk Factors for COVID-19 Severity and ACE2 Expression

ACE2 is regulated by a gene which maps on the X chromosome (Xp22.2), thus suggesting that some differences may exist in the expression of ACE2 between men and women (25). In women to prevent the redundant expression of the products of the genes present in double copy on the X chromosomes, a physiological random inactivation occurs in one of the two chromosomes (25). The remained chromosomal portions that escape to the inactivation and the genes present in these areas (~15%) can be over-expressed in women (25). ACE2 is encoded precisely in these regions of the X chromosome which escape the inactivation of one of the two X chromosomes, supporting the hypothesis of a greater ACE2 expression in women (25). There is evidence that ACE2 tissue levels are also regulated by estrogens that can increase the presence of ACE2 receptor (26). Thus, if, as reported by several commentary in literature, the presence of ACE2 throughout the body could make tissues more vulnerable to SARS-Cov-2 infection women should be more predisposed to the virus than men (26). On the contrary, epidemiological data of the World Health Organization (WHO) highlighted gender-based clinical differences in SARS-CoV-2, with a higher mortality rates in male patients, in particular elderly patients (27). Even this latest information appears to be in contrast with the hypothesis that ACE2 throughout the body could make tissues more vulnerable to SARS-Cov-2 infection. In fact, it was demonstrated that ACE2 level decrease with age and seem to be higher in young people that commonly develop a less severe COVID-19 form (26). It is important to underline that also the opposed hypothesis, that a mild/moderate ACE2 deficiency may protect from SARS-CoV-2 invasion, seems improbable considering the high affinity of the virus for ACE2 receptor. In addition, this latter hypothesis is also unlikely because different degree of ACE2 deficiency are related with specific diseases, i.e., diabetes, obesity and cardiovascular disease, that characterize individuals more prone to be infected and to have severe complications related to SARS-CoV-2. These inconsistencies highlight that other factors, such as for example organ-specific ACE2 distribution and expression levels and potential co-expression and interaction with specific proteases, may contribute to the severity of SARS-CoV-2.

Although it is demonstrated that lungs inflammation is one of the main symptom during SARS-CoV-2 infection, the lungs, among all organs, present a moderate expression of ACE2 and, as reported above, SARS-CoV-2 may affect other organs, organs that have a high to moderate expression of ACE2. In this context a detailed map of the physiological organ-specific distribution, expression, and activities of ACE2, also considering organ-specific gender biases and organs often poorly considered (specific brain regions, oral cavity, thyroid, pancreas, duodenum, colon, rectum, gallbladder, male -testis and seminal vesicle- and female tissues -ovary, oocyte, uterus, vagina-, skin, and others), and a complete overview on the potential link between these organs and SARS-CoV-2 may contribute to understand the potential infection routes as well as the clinical symptoms and mechanisms of the virus susceptibility.

## ACE2 in Human Physiology: Body Localization, Expression, Function and Activities

**Figure 1:**
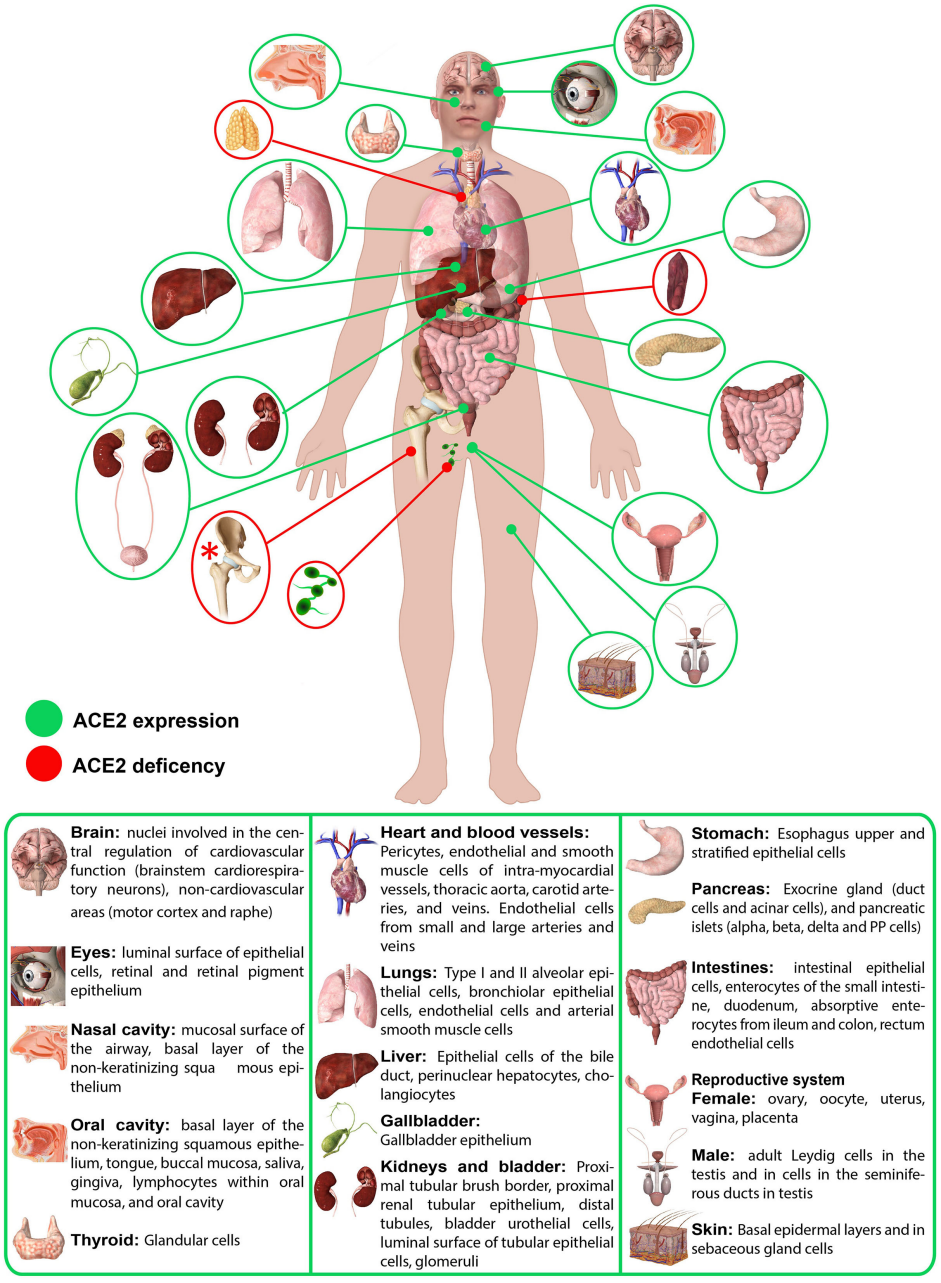
Schematic representation of ACE2 expression in human organs. ACE2 mRNA is present in all organs (28). ACE2 protein expression is present in heart, kidney, testis, lung (type I and type II alveolar epithelial cells), nasal, and oral mucosa and nasopharynx (basal layer of the non-keratinizing squamous epithelium), smooth muscle cells and endothelium of vessels from stomach, small intestine and colon, in smooth muscle cells of the muscularis mucosae and the muscularis propria, in enterocytes of all parts of the small intestine including the duodenum, jejunum, and ileum (but colon), skin (basal cell layer of the epidermis extending to the basal cell layer of hair follicles smooth muscle cells surrounding the sebaceous glands, cells of the eccrine glands), endothelial, and smooth muscle cell of the brain (28). Red asterisk (*): ACE2 deficiency only hypothesized.

About 20 years ago, the first paper reported the mapping of ACE2 in 72 tissues (28). Over the years, it has become more and more clear that ACE2 localization can be quite tricky (28). Starting from the localization in the renal and cardiovascular tissues, over time it has become evident that ACE2 is also present in tissues and organs where initially no trace of it was detected (Figure 1), as in the gastrointestinal tract, up to recent studies that report slight positivity even in locations so far considered ACE2 free, such as in circulating leukocytes (29–31).

There is no question that the ACE2 receptor is also expressed at the level of epithelia of the respiratory system (tracheal and bronchial epithelial cells, alveolar epithelial cells, type 2 pneumocytes), cardiovascular system (endothelium of coronary arteries, myocites, epicardial adipocites, vascular endothelial, and smooth cells), gastrointestinal tract (esophagus keratinocytes, gastrointestinal epithelial cells, intestinal epithelial cells, duodenum, small intestine, rectum), urogenital system (kidney proximal tubules, bladder urothelial cells, luminal surface of tubular epithelial cells, testis, seminal vesicle), as well as in the liver and gallbladder and in the nervous system. (25, 28, 32) However, it is important to underline that, while mRNA seems to be expressed homogeneously in all tissues, the same is not always certainly for protein expression (Figure 1) (28).

Many studies over the years have focused on the role of ACE2 in the cardiovascular system, both for the functions of the renin–angiotensin system (RAS) system and for the study of new therapeutic targets in cardiac pathologies (15, 16). ACE2 is recognized as a protector of vascular tissues, balancing angiotensin II effects, protecting endothelia, and promoting mechanisms of regeneration (15). Intuitively, ACE2 impairments leads to severe cardiac dysfunction, with increased atherosclerosis, and endothelial damage. ACE2 is studied also in hypertension models, as genetic variation affects systolic function in men and ventricular mass in women (33). Not by chance, increased levels of ACE2, both at the gene level and protein expression, but also in circulating soluble forms, are detected after myocardial injury, suggesting a potential role as cardiac biomarker (15). Cardiac alterations result to be usually correlated with thyroid dysfunction, particularly to hyperthyroidism (34). Thyroid hormones also seem to act on ACE2 expression both influencing the receptor gene expression and conditioning the release of ACE from lung endothelium (35). ACE2 has been investigated also as cancer marker, as it has been observed an increase of ACE2 expression in thyroid cancer with an increase of ACE2/ACE *ratio* proportional to the differentiation grade of the cancer (36). The activity of ACE2 in cardiovascular system is strictly related to those in brain, as ACE2 is expressed in the neuronal area deputy to cardiovascular control, so that is result to be less expressed in case of cardiac injury, while an over-expression in brain leads to a protective action, *via* reduction of pro-inflammatory cytokines and augmentation of NO activity (37). Many animal models have been used for the study of ACE2 role in the brain. Data highlighted the antihypertensive and sympatholytic action of ACE2 in the hypothalamus *via* reduction in Ang II and increase in Ang-(1–7) levels, and a positive effect of ACE2 in the neuronal recovery from stroke (38). ACE2 is also involved in mechanism of memory, *via* regulation of brain-derived neurotrophic factor expression, and the production of reactive oxygen species, in stress response, regulating corticotropin releasing hormone at hypothalamus level, and in neurogenesis related to serotonin level, secondary to the availability of its precursor tryptophan (39, 40). The fil rouge between tryptophan synthesis and ACE2 crosses the activity in many systems and binds their functionality. In fact, ACE2, involved in the RAS mediated homeostasis, plays at intestinal level regulating the microbiome, acting on amino acid uptakes, and expression of antimicrobial peptides (41). ACE2 acts as amino acid transporter, binding B0AT1, a neutral amino acid transporter, in the small intestine, and in animal model of ACE2 deficiency a reduction of tryptophan levels in the blood has been demonstrated (42). This reduction is reflected in the intestine with greater inflammation at the level of the colon, endothelium alteration and reduced ability to damage response, involving also mammalian target of rapamycin (mTOR) pathway, a member of the phosphatidylinositol 3-kinase-related kinase family of protein kinases (43). During the years attention was addressed also to the intestine, since it is known to express the highest level of ACE2 (28). In addition to ACE2 localization in the intestine, ACE2 was found in smooth muscle cells and endothelium of vessels from the stomach, and colon, smooth muscle cells of the muscularis mucosae, and the muscularis propria (28). ACE2 was also copiously present in the enterocytes of all parts of the small intestine including the duodenum, jejunum, and ileum, but not in enterocytes of the colon (28). In addition to the gastrointestinal tract, ACE2 has been found in kidney and in pancreas, with dislocation similar to those of ACE2 that is in kidney apical surface area of the proximal tubules and pancreatic acini and islets (28). As for pancreas, the presence of ACE2 influences islets status *via* regulation of blood pressure and NO release, as well as acting on tissue fibrosis (44). The role of ACE2 has been widely investigated also for the onset of diabetes, as ACE2 deficiency has been associated with impairment of first-phase insulin secretion and of glucose tolerance (45–47). The alteration of RAS system and specifically of ACE2 activity induces an alteration of pancreatic islets, due to unbalance NO production, which in turn influences blood flow, also secondary to glucose availability (45–47). The wide expression of ACE2 in kidney is not so surprisingly, considering the pivotal role of RAS system in this organ, in which it regulates the electrolytic equilibrium *via* reabsorption of sodium and water into the blood, while causing excretion of potassium (48). ACE2 acts balancing the RAS activity, regulating renal homeostasis and it is postulated that its activity is more related to a local control than a systemic regulation of blood pressure (48). Effects of reduced ACE2 are described as promoting proteinuria, in particular albuminuria, glomerular disease and are related to diabetic nephropathy, with lower ACE2 expression at tubular level (48). Despite the role of ACE2 in hepatic glucose metabolism is not completely investigated, the alteration of the ACE2 pathway is, also in this localization, related to the development of impairment of metabolic activity, and in particular of insulin resistance (48). ACE2 in liver has been found expressed in endothelial cells, bile duct cells, and perinuclear hepatocytes and it was mostly elevated in hepatic fibrogenic resistance (28). Notably, insulin resistance correlates with endothelium-dependent and insulin-mediated vasodilatation (46, 49). In addition, a recent RNA-seq data in the human protein atlas database have shown the highest expression of ACE2 in liver cholangiocytes, followed by hepatocytes (50)

ACE2 expression seems to be correlated to the sensory organs. However, the real expression of ACE2 at ocular level, instead, seems to be still object of debate. Although it is the least widely expressed among the RAS system components, ACE2 is detectable in the aqueous humor (51, 52). Some papers declare a not significant mRNA presence and immunoreactivity of ACE2 in human conjunctiva (53), while according to others, ACE2 gene expression is detectable both in human conjunctiva and primary pterygium tissues, even if in a reduced cohort of patient (54). ACE2 is expressed at the oral level in particular at the oral tongue than at the buccal and gingival and could be found in epithelial cells, T cells (<0.5%), B cells (<0.5%), and fibroblast (<0.5%) (31). In addition, in the oral and nasal mucosa and in the nasopharynx, ACE2 expression was found in the basal layer of the non-keratinizing squamous epithelium (55). Human ACE2 was detected in ciliated airway epithelial cells of human airway tissues derived from nasal regions (55). Concerning ACE2 presence at the ear level no data were present on human. However, a recent online study found high expression of ACE2 in the cat ear tip (56). Another sensory organ where ACE2 was also found is at the skin level (57). The activity of RAS system in controlling cell proliferation and differentiation, also in case of tissues injury in the mechanism of self-renewed of damaged cells and neo-angiogenesis, is reflected also in the skin, where the epidermal stem cells express the different players of this system, including ACE2 (57). Immunohistochemical evaluation of ACE2 presence in healthy and oncologic patients showed ACE2 in basal cell layer of normal epidermis and sebaceous glands and a reduction of ACE2 reactivity in patients affected by pre-malignant lesions (actinic keratosis) and non- melanoma malignant skin cancers (basal cell carcinoma and squamous cell carcinoma), suggesting an involvement in the pathogenesis of the disease (58).

Considering the role of Angiotensin II in the menstrual cycle, the presence of ACE2 in the female reproductive systems appear quite intuitive. In fact, AngII acts on follicular, ovulatory and luteinic phases, influencing follicle development, oocytes maturation, and corpus luteum progression, balancing the levels of steroid hormones (59). In addition, it promotes spiral artery vasoconstriction and endometrium regeneration at the uterus level. Angiotensin II has been identified also as a player in endometrium fibrosis and endometrial metastases (59). Not surprisingly, during pregnancy, ACE2/AngII/Ang 1–7 axis is involved in maintenance of blood pressure and alterations of this pathway correlate with disorders like pre-eclampsia and eclampsia, while reduction of ACE2 expression negatively influences gestation and fetus birth (60). In parallel, ACE2 expression has been detected also in testis, particularly in spermatogonium and Leydig and Sertoli cells, with possible correlation with spermatogenesis and maintenance of functional and structural integrity of the apparatus (61).

Finally, despite the presence of ACE2 in numerous organs, tissues and cells have not been completely clarified and in many of them not yet investigated, ACE2 seems to be absent in the spleen, thymus, lymph nodes, bone marrow, and in several cells of the immune system (15, 62). However, it is important to underlined that numerous studies on ACE2 expression in bone marrow are currently in progress since all the players of RAS system are present in the bone marrow, acting on cell lineages proliferation and also in hematopoietic restoration after myelosuppression, and ACE2 seems to have in particular a role in CD34+ proliferation (63).

In this moment, with the ongoing COVID-19 pandemic, this rapid overview related to the distribution, expression and activities of the ACE2 in human body could help and improve our understanding on potential infection routes of SARS-CoV-2 through the body. Thus, in the next section we discuss how the presence, distribution and abundance of ACE2 in specific target organs may be related to the COVID-19 clinical symptoms and manifestations.

## SARS-COV-2 Clinical Implication and Potential Mutual Interactions With ACE2

### Nasal Cavity

On October 5, 2020 searching on PubMed “COVID-19 OR COVID-2019 OR severe acute respiratory syndrome coronavirus 2 OR severe acute respiratory syndrome coronavirus 2 OR 2019-nCoV OR SARS-CoV-2 OR 2019nCoV OR (Wuhan AND coronavirus) AND (Nose OR Nasal Cavity)” we found 388 papers. Most of the studies were guidelines on how to perform nasal and oropharyngeal swab procedure for the screening of COVID-19 infection. The other studies detected, analyzed and discussed the different nasal manifestations in COVID-19 patients (64). Orhinolaryngological symptoms resulted common manifestations of COVID-19, particularly in mild or moderate form of the disease (65, 66). The nasal cavity and turbinates have important physiological functions in filtering, warming, and humidifying inhaled air and these functions are critical during SARS-CoV-2 infection since the nasal cavity is the principal gateway for virus entrance. In fact, epithelial cells in this region are considered suitable clinical sample for early virus detection. Increasing number of reports on SARS-CoV-2 positive patients described olfactory dysfunction, such as loss of smell, cacosmia, phantosmia nasal obstruction or rhinorrhea, and nasal congestion (1, 67– 73)^1^. Some data also reported SARS-CoV-2 positive patients with isolated anosmia, without any other symptoms, suggesting these patients as a potential source of rapid virus spread (68, 69, 74). Anosmia in SARS-CoV-2 positive patients can be present as primary symptom or as an early symptom, with different percentage among the examined studies, percentages that can range from 6 to ~80% (1, 70–73, 75). Lechien et al. demonstrates that about 87% patients with an anosmia duration ≤ 12 days were also PCR SARS-CoV-2 positive (73). Additionally, Kaye et al. analyzing a court of 237 COVID-19 patients showed that 73% of patients reported anosmia and 26.6% reported loss of sense of smell as initial symptom (70). Patients below 40 years, particularly female, seem to be the more prone to develop SARS-CoV-2 form with only hyposmia/anosmia manifestations (1, 69–72). However, an Asian study reported a lower percentage of patients with olfactory dysfunctions in comparison to European patients (73). This aspect may be probably due to the diverse ACE2 polymorphisms and expression level between Asian and European individuals (74). The loss of smell in SARS-CoV-2 patients may be caused by different factors, such as localized olfactory cleft oedema, architectural deformity of the olfactory neuroepithelium, or direct neuro-invasion of the olfactory nerve pathways. As above described, it is important to underline that gene expression databases highlighted a moderately/high expression of ACE2 in human olfactory mucosa (76). However, to date, whether ACE2 expression in the olfactory epithelium is neuronal or non-neuronal or if it occurs in both cell types it is not completely clear (77, 78). SARS-CoV-2 brain infection could be facilitated by the neuronal expression of the host receptors through absorption in ciliated dendrites/soma and consequent axonal anterograde transport along the olfactory nerve (79, 80). Concerning the non-neuronal expression of ACE2, it could be due to the nasal cavity olfactory epithelium that would work as virus reservoir (79, 80). Several RNaseq transcriptome reports conducted in human and murine olfactory epithelium suggested a non-neuronal expression of ACE2 as well as of TMPRSS2 (79–82), but further studies are mandatory to confirm these finding. It was also shown that nasal brushing epithelial cells, nasal turbinate epithelial cells, and nasal airway epithelial cells contained ACE2-expressed and TMPRSS2-expressed cell clusters (82).

### Oral Cavity

On October 5, 2020 searching on PubMed “COVID-19 OR COVID-2019 OR severe acute respiratory syndrome coronavirus 2 OR severe acute respiratory syndrome coronavirus 2 OR 2019-nCoV OR SARS-CoV-2 OR 2019nCoV OR (Wuhan AND coronavirus) AND oral cavity” we found 218 papers. Several studies evaluated the presence of SARS-CoV-2 in saliva through entry into the oral cavity with several potential pathways, *via* a direct infection of oral mucosa lining cells, *via* droplets from the respiratory tract, from the blood circulation by gingival crevicular fluid, or *via* extracellular vesicles released from infected cells and tissues (83). To et al. confirmed that SARS-CoV-2 can be detected by PCR in about 92% of saliva samples, indicating the saliva as a potential source of SARS-CoV-2 spreading (84). The presence of SARS-CoV-2 in patients' saliva also suggested the likelihood of salivary gland infection. Chen et al. collecting saliva directly from salivary gland, found SARS-CoV-2 nucleic acid, hypothesizing that salivary glands were SARS-CoV-2 infected (85). This hypothesis is further reinforced by the fact that the ACE2 epithelial cells of the salivary glands have been shown to be an early target for the SARS-CoV-2 (86). In addition, high levels of mRNA and protein levels of the cellular protease Furin as well as of TMPRSS2 have also been found in the salivary glands (86). Thus, the possible role and function of salivary gland cells in the initial SARS-CoV-2 entry and progress must be further considered and validated as well as their potential function as virus reservoir, able to establish a persistent infection which could last also for months (87). Furthermore, it should be underlined that the saliva samples not only contain saliva secreted from the salivary glands but also the secretions from the nasopharynx and from the lung *via* the action of cilia lining the airway. Thus, more studies are needed to delineate the real sources and functions of SARS-CoV-2 in saliva.

Another point related to the oral cavity is represented by the fact that numerous studies reported an acute loss of taste (hypogeusia/ageusia) as a frequent symptom of SARS-CoV-2 infection, particularly common among females and younger individuals (~20–39 years) (1, 84, 85). A recent case series presented several cases of SARS-Cov-2 infection where the loss of taste was also associated with oral lesions (88). These lesions presented two distinct patterns, one resembling aphthous-like ulcers in young patients with mild cases of COVID-19 and another with more widespread patterns resembling Herpes Simplex Virus 1 necrotic ulcers in the more severe and immunosuppressed older individuals (89). Whether these lesions were due directly by SARS-CoV-2 or were an associated manifestation resulting from the severe compromised state of the patient remains to be determined. However, what is known is that taste disorders are linked to an extensive variety of viral infections (90). Upper respiratory tract infection can lead to acute onset ageusia because of viral damage to the olfactory epithelium (90). Furthermore, as previously reported for the nasal cavity, viruses can also use the olfactory nerve as a route into the central nervous system (CNS). Thus, ageusia may be a secondary result of olfactory dysfunction. However, it is important to underline that ACE2 is not only extensively express in the salivary glands and in oral tissues and its expression was higher in tongue than buccal or gingival tissues (https://gtexportal.org). Furthermore, ACE2 positive cells were enriched in epithelial cells, thus damage of mucosal epithelial cells of the oral cavity may explain ageusia, oral mucosal ulcerations, and necrosis detected in SARS-CoV-2 patients (31, 91). In addition, it was reported that the ACE2 within oral mucosa is also expressed in lymphocytes, and comparable results were also reported for other organs of the digestive system (31). However, since the ACE2-positive lymphocytes is quite few whether this aspect could indicate that SARS-CoV-2 attacks the lymphocytes and leads to the severe disease in patients' needs further studies (31). More in generally SARS-CoV-2-mediated gustatory disturbances has not yet been definitively identified.

### Eyes

On October 5, 2020 searching on PubMed “COVID-19 OR COVID-2019 OR severe acute respiratory syndrome coronavirus 2 OR severe acute respiratory syndrome coronavirus 2 OR 2019-nCoV OR SARS-CoV-2 OR 2019nCoV OR (Wuhan AND coronavirus) AND (eyes OR ocular manifestations)” we found 820 papers. Most of the studies were official recommendations of ophthalmological societies for precaution and prevention of SARS-CoV-2 infection or studies on the impact of COVID-19 outbreak on eye care. Currently, the presence and prevalence of ocular manifestations in SARS-CoV-2 infection, consistent with conjunctivitis and including conjunctival hyperemia, chemosis, epiphora, or increased secretions, are still controversial (92–95). Despite it was reported that only a small percentage (from about 1 to 6%) of SARS-CoV-2 positive patients developed signs of conjunctivitis, other studies showed that up to 31% of SARS-CoV-2 hospitalized patients presented conjunctivitis (96–99). Wu et al. showed that about 31.6% of COVID-19 patients had ocular abnormalities, and ocular symptoms were more frequent in severe cases of COVID-19 patients (97). In fact, about 50% of COVID-19 patients with ocular abnormalities were classified as critical, 16.7% were classified as severe, and 33.3% were classified as moderate severity (97). In addition to conjunctivitis other ocular abnormalities directly correlated with the COVID-19 severity seem to be alterations in retina and in its vasculature (100). A recent study evaluating the retina of patients with COVID-19, within 30 days from the onset of systemic symptoms, found an enlargement of retinal arteries and veins in more severe cases and showed an inverse correlation with time to symptoms onset (100). In this context, Casagrande et al. demonstrate the existence of SARS-CoV-2 nucleic acid in the human retina COVID-19 patients (101). Additional studies also highlighted the presence of SARS-CoV-2 RNA in tear film and/or conjunctival swabs of COVID-19 patients with conjunctivitis but not in patients without ocular symptoms (98, 102–105). Differently, Xie et al. demonstrated that the SARS-CoV-2 RNA was present also in the normal ocular surface of COVID-19 patients without conjunctivitis (106). Despite this point is still debated, it is critical to underline that ocular surfaces have a great tropism for respiratory viruses and also for coronavirus (107, 108). Whether specifically SARS-CoV-2 may infect retina and conjunctival cells in human remains unclear. Based on the current literature, several reports hypothesized that the exposure of the ocular surface to SARS-CoV-2 could lead to infection probably due to the drainage of virus particles *via* the nasolacrimal duct, specifically through the lacrimal canaliculi that drain tears from the eye surface into the nasal cavity, into the respiratory tract (109, 110). In this context it is important to emphasized that others reports also considered the presence of ACE2 and TMPRSS2 on the cornea and conjunctiva as a possible virus route (56, 111, 112). The presence of the ACE2 and TMPRSS2 on the corneal cells may allow the virus to cross the ocular surface, and then spread from the eye to other parts of the body through the blood stream and/or through the nervous system (ophthalmic branch of trigeminal nerve) (112, 113). However, to date, there are no clear evidences that SARS-CoV-2 virus, in humans, can enter inside the eye or spread to the brain through corneal nerves (114).

### Lungs

On October 5, 2020 searching on PubMed “COVID-19 OR COVID-2019 OR severe acute respiratory syndrome coronavirus 2 OR severe acute respiratory syndrome coronavirus 2 OR 2019-nCoV OR SARS-CoV-2 OR 2019nCoV OR (Wuhan AND coronavirus) AND lungs” we found 4,138 papers. While SARS-CoV-2 was detected in many organ systems, the lungs seems to be the main organs affected by the virus (96, 115). In fact, it is known that the upper respiratory tract and lungs serve as predominant site of virus entry and replication and that SARS-CoV-2 patients showed the symptoms of pneumonia and alveolar damage (116). The most common and severe complication in patients with SARS-CoV-2 patients is acute hypoxemic respiratory failure or acute respiratory distress syndrome that lead to oxygen and ventilation therapies (1, 117–135). Some of these critically ill patients also required intubation and invasive ventilation. Lungs radiological images and computed tomography (CT) scans of SARS-CoV-2 positive patients provided numerous information about the severity of the infection and showed abnormal results in about 86% of patients (1, 117–135). The most common patterns of radiological images and CT scans were ground-glass opacities, consolidation, centrilobular nodules, architectural distortion, bronchial wall thickening, vascular enlargement, traction bronchiectasis, reticulation, crazy paving pattern, intrathoracic lymph node enlargement, and subpleural bands, that cause pulmonary discomfort and require rapid diagnosis and treatment (1, 117–135). In addition, autoptic results revealed that in about 48% of cases the predominant histopathological finding were capillary congestion, microthrombi as well as moderate intra-alveolar fibrin exudation resultant in exudative disseminate alveolar damage and superimposed bronchopneumonia (135). A more widespread histological pattern of alveolar damage with greater fibrotic evolution in the lungs was observed in patients who died after a long period of mechanical ventilation (136). In few cases, an intra-alveolar deposition of neutrophilic granulocytes, probably due to superimposed bacterial infection, was also detected (137). Since the distribution of ACE2 in different organs seems to be notably linked to the clinical symptoms of SARS-CoV-2 infection and since the acute respiratory distress syndrome is a potential deadly complication of SARS-CoV-2, research studying lung complications of ACE2 down-regulation are of key significance in this context. Several studies on lung injury highlighted that ACE2 receptors down-regulation lead to critical inflammatory lesions in the respiratory tract (alveolar wall thickening, edema, infiltrates of inflammatory cells, bleeding) which seem to be carried out by angiotensin II (135, 138–142). A key point to remark is that the wide surface of alveolar epithelial cells might explain the vulnerability of lungs to the virus invasion. As previously explained ACE2 are principally expressed in type II pneumocytes, little cylindrical cells that correspond to the 5% of all pneumocytes (1). These type of pneumocytes exert immunoregulatory functions and are of key importance for alveolar surfactant production and they also function as stem cells, progenitors of type I pneumocytes, that represent the 95% of all pneumocytes and that are responsible of gas exchanges (142). Thus, the damage of type II pneumocytes owing to the binding of SARS-CoV-2 to ACE2 receptors is critical for several factors, i.e., for the local unopposed ACE → Angiotensin II → AT1 receptor axis over-activity, for the reduced production of alveolar surfactant by injured type II pneumocytes that lead to reduced lung elasticity and, finally, for the reduced repair of type I pneumocytes that bring to impaired gas exchanges and fibrosis (143). While ACE2 is expressed in the bronchial epithelium and in type 2 pneumocytes, TMPRSS2 results strongly expressed in the cytoplasm of bronchioles and alveolar epithelial cells (144). Since ACE2 was found to exist on alveolar epithelial cells at approximately similar level as in the whole lung, Sato et al. found that the expression level of TMPRSS2 was considerably different between the peripheral and central parts of the lung (145). Thus, since that peripheral parts of the lung strongly express TMPRSS2, along with ACE2, the SARS-CoV-2 may be considered to damage the peripheral area at the beginning of infection. These data explain why chest CT revealed consolidation and ground glass opacities in the bilateral peripheral lobes in COVID-19 cases (146). However, these factors would not even prevent the simultaneous role of other mechanisms including an altered immune response to initial viral invasion, or a genetic susceptibility to hyper-inflammation and thrombosis (8, 147). In SARS-CoV-2 pneumonia, thrombosis may play a direct, and critical role in gas exchange abnormalities and in multisystem organ dysfunction. Unfortunately, to date, as for all the other organs affected by SARS-CoV-2, the lungs impairment during this new infection remain to be further clarified.

### Heart and Blood Vessels

On October 5, 2020 searching on PubMed “COVID-19 OR COVID-2019 OR severe acute respiratory syndrome coronavirus 2 OR severe acute respiratory syndrome coronavirus 2 OR 2019-nCoV OR SARS-CoV-2 OR 2019nCoV OR (Wuhan AND coronavirus) AND (cardiovascular system OR heart OR blood vessels)” we found 3,170 papers. In most of these reports cardiovascular complications emerged among the most significant manifestations in SARS-CoV-2 infection (148–155). Different cardiovascular complications, such as myocarditis, acute coronary syndrome, decompensated heart failure, pulmonary embolism, cardiogenic shock, and infection of a heart transplant recipient, accompanied by altered levels of creatine kinase isoenzyme-MB, myohemoglobin, cardiac troponin I, and N-terminal pro-brain natriuretic peptide were currently reported (1, 149–155). In addition, a high prevalence of pre-existing cardiovascular morbidities, including hypertension, and coronary artery diseases, has been detected among patients with severe SARS-CoV-2 (1, 149, 156). In COVID-19 patients, the highest mortality rates were also observed in case of pre-existing cardiovascular disease and elevated cardiac troponin levels (137, 157). Furthermore, patients with higher troponin levels had also increased markers of inflammation, including C-reactive protein, interleukin (IL)-6, ferritin, lactate dehydrogenase (LDH), high neutrophil count, and high amino-terminal pro-B–type natriuretic peptide (158). Despite it was initially hypothesized that COVID-19 patients with pre-existing cardiovascular morbidities and treated with ACE inhibitors (ACEi) or angiotensin receptor blockers (ARBs) (155, 159, 160). could be at increased risk for severe SARS-CoV-2 infection, a recent retrospective study on COVID-19 patients with hypertension showed that ACEI/ARB therapy attenuated the inflammatory response (161). In addition, a study on SARS-CoV-2 patients with hypertension showed no difference in the percentage of patients treated with ACEi/ARBs between those with severe and non-severe infection and between survivors and non-survivors (162). However, understanding the positive or negative effect of ACEi/ARB in COVID-19 appears to be complex, and this could also be due to the clinical stage of the virus (viral contamination phase vs. tissue inflammation phase). Several clinical trials on this question are forthcoming (NCT04329195, NCT04331574, NCT04351581, NCT04353596). To date, the mechanisms by which SARS-CoV-2 leads to cardiac manifestations is currently unclear. These mechanisms would involve several factors such as a direct viral damage and an immune-mediated damage by inflammatory cytokines (i.e., a systemic cardiotoxic cytokine-storm), and cytotoxic immune cell response. As reported in the previous section, the cardiac tissue presents a high ACE2 expression level (163). Specifically, it was shown that cardiomyocytes from the heart contain about 6% ACE2-expressed cells and 0.8% TMPRSS2-expressed cells, and the cardiovascular progenitor cells contain 12.5% ACE2-expressed cells and 0.4% TMPRSS2-expressed cells, thus SARS-CoV-2 could directly infect the myocardial tissue (82). In addition, Furin can also be considered a critical molecule that makes SARS-CoV-2 cause adverse cardiovascular events through the ACE2 receptor. This speculation is supported by the occurrence of high level of Furin in the peripheral blood of COVID-19 patients (164). Additionally, PCR analyses also identified SARS-CoV-2 in the cardiac tissue of ~35% of infected patients, further supporting that a direct viral damage can occur (165). Kuba et al. by using a mouse model showed that SARS-CoV pulmonary infection leads to an ACE2-dependent myocardial infection (138). This infection can lead to a localized inflammatory response with resulting myocarditis that bring to acute cardiac injury and the prospective for arrythmias or heart failure (166). Autoptic data on SARS-CoV-2 positive patients showed the existence of mononuclear inflammatory myocardial infiltrate, thus supporting this hypothesis also for this new coronavirus (3). Numerous studies also reported immunological derangements in SARS-CoV-2 positive patients (167, 168). This altered immunologic status has been related with an increased risk of cardiovascular disease and could be also an indirect mechanism of immunological dysfunction that lead to cardiac sequelae (166–168). In addition, numerous SARS-CoV-2 positive patients showed respiratory distress that lead to hypoxemia that could cause cardiac injury secondary to an oxygen mismatch (166–168). Other systemic consequences of cardiac injury in SARS-CoV-2 patients could also be related to sepsis and disseminated intravascular coagulation (DIC) that vary from minimal change to interstitial inflammatory infiltration and myocyte necrosis vasculature microthrombosis and vascular inflammation (166–168). However, to date whether SARS-CoV-2 infection impair the heart remains to be further demonstrated.

### Kidney and Bladder

On October 6, 2020 searching on PubMed “COVID-19 OR COVID-2019 OR severe acute respiratory syndrome coronavirus 2 OR severe acute respiratory syndrome coronavirus 2 OR 2019-nCoV OR SARS-CoV-2 OR 2019nCoV OR (Wuhan AND coronavirus) AND (kidney OR urinary system)” we found 1,031 papers. The kidney is one of the major organs which play a key role in the filters which excrete toxins, waste products, and extra water from our body. Despite most of the work were focused on kidney transplantation and on the management of dialysis patients during SARS-CoV-2 infection, several studies reported an increased incidence of acute renal injury during the infection (169, 170). The bladder may also be affected and may ultimately lead to multiple-organ failure and death (169, 170). Although initial reports suggested that the burden of acute kidney injury during SARS-CoV-2 infection was moderately low (about 0.5%), recent studies reported an incidence going up to 56.9% (115, 169, 171–174). In critically ill patients, this incidence was remarkably higher, ranging from 61 to 76% (175). A higher incidence of acute renal injury has been reported in USA and UK than in China (96, 150, 174, 176). Several studies also showed that patients with acute renal injury have a higher mortality rate compared to other patients and this is particularly true for those in the ICU (177–179). In a recent study, it was shown a high incidence of renal dysfunction (46%) and acute renal injury (29%) also in hospitalized children with COVID-19 (180). Patients with acute renal injury also showed elevated levels of serum creatinine and blood urea nitrogen associated to higher leukocyte count and lower lymphocyte and platelet counts (169). Prolonged activated partial thromboplastin time and higher D-dimer, both coagulation parameters, were also more common in these patients (169). In addition, a high percentage of SARS-CoV-2 patients with acute renal injury had proteinuria albuminuria and hematuria, along with isolation of viral RNA from urine, all factors that support the potential viral tropism for the kidney (181, 182). This tropism was also confirmed from an autopsy study by Su et al. that demonstrated by electron microscopy SARS-CoV-2 presence in the renal tubular epithelium of seven of 26 SARS-CoV-2 patients (176). This study also showed the presence of a diffuse proximal tubule injury with the loss of brush border, non-isometric vacuolar degeneration, necrosis, and occasionally hemosiderin granules and pigmented casts (176). In addition, a prominent erythrocyte aggregates obstructing the lumen of capillaries without platelet or fibrinoid material were also detected (176). Clusters of coronavirus-like particles with distinctive spikes in the tubular epithelium and podocytes were also detected. Post-mortem examination of the viral nucleocapsid protein *in situ* in the kidney also showed that SARS-CoV-2 antigens is accumulated in kidney tubules, suggesting that SARS-CoV-2 may infects kidney directly, leading to acute renal injury and potentially contributing to viral spread (183–185). This direct route of SARS-CoV-2 may be due to an ACE2-dependent pathway. It was found that both proximal tubular cells or tubular progenitor cells in the kidney co-expressed ACE2 and TMPRSS2 and their expression levels resulted high in nephron epithelial cells, epithelial cells, endothelial cells, and mesangial cells of the kidney (82, 186). Additionally, Pan et al. showed that the TMPRSS2 gene was co-expressed with ACE2 in kidney podocytes (170). These cells are particularly vulnerable to viral infection and their injury easily induces heavy proteinuria that was detected in about 43.9% of SARS-CoV-2-infected patients (181). The co-expression of ACE2 and TMPRSS2 in renal tubular cells could imply that SARS-CoV-2 may directly bind to ACE2-positive cells in the kidney and destroy the function of renal tubules. However, kidney disease involvement in SARS-CoV-2 patients is likely to be multifactorial and may be also due to cytokine damage (high levels of IL-6), organ crosstalk (Lung-kidney) and other systemic effects (187, 188).

### Stomach and Intestines

On October 6, 2020 searching on PubMed “COVID-19 OR COVID-2019 OR severe acute respiratory syndrome coronavirus 2 OR severe acute respiratory syndrome coronavirus 2 OR 2019-nCoV OR SARS-CoV-2 OR 2019nCoV OR (Wuhan AND coronavirus) AND (stomach OR intestines OR gastrointestinal system OR digestive system)” we found 977 papers. A lot of studies showed that the gastrointestinal tract represents a common target organ of SARS-CoV-2 infection (1, 2, 29, 96, 150–152, 189–198). A recent study suggests that the gastrointestinal symptoms in COVID-19 patients can be present up to 50% (39.6–50%), with symptoms including nausea, diarrhea, anorexia, abdominal pain, belching, and emesis (199, 200). Anorexia appears to be the most common gastrointestinal symptom (26.8%), but the mechanism of its onset in COVID-19 patients remains unclear (4). However, this symptom can be due to gustatory dysfunction, which was found in a high percentage of COVID-19 patients (201). Several data reported that these gastrointestinal manifestations during SARS-CoV-2 infection can be associated with a poor disease course; comparing patients with non-severe disease with those with severe infection it was shown a higher risk of developing gastrointestinal symptoms in patient with severe infection (29, 202). The occurrence of these gastrointestinal symptoms can not only coexist with other symptoms, but also precedes the typical phenotype of SARS-CoV-2 infection (203). It was shown that also pediatric patients and children with SARS-CoV-2 infection may present digestive symptoms, most commonly diarrhea, in the absence of respiratory symptomatology (203, 204). Although different clinical features, such as milder disease course symptoms are present in pediatric patients and children with SARS-CoV-2, the gastrointestinal symptoms appear to be similar to those found in adult individuals (204). Despite gastrointestinal symptoms were frequently observed in SARS-CoV-2 patients, to date, the exact significance of these manifestations are still unclear. An autopsy report, with details of gastrointestinal pathology in a SARS-CoV-2 patient, showed the presence of segmental dilatation and stenosis in the small intestine (205). To date, autopsy data and reports with a full description of the gastrointestinal appearance associated to SARS-CoV-2 infection are still few to allow a clear conclusion. In addition to the clinical symptoms induced by the gastrointestinal disorders during SARS-CoV-2 infection, these manifestations can highlight one more route of virus transmission, i.e., the fecal-oral transmission. An increasing number of data showed that stool samples contain high concentration of SARS-CoV-2 RNA during infection for a relatively long period of time (from 1 to 12 days) (193, 204, 206). These data were also confirmed in pediatric patients and in children where ~80% of patients resulted positive on rectal swabs even after negative nasopharyngeal tests (204). This aspect suggests a potential replication of SARS-CoV-2 virus in the gastrointestinal tract. This hypothesis is partially confirmed by Lin et al. that analyzing by endoscopy severe and non-severe SARS-CoV-2 patients with gastrointestinal manifestations detected the presence of SARS-CoV-2 RNA in esophagus, stomach, duodenum, and rectum of severe patients while only in the duodenum on one of four non-severe patients (4). Although, there are numerous data on gastrointestinal symptoms during SARS-CoV-2, the exact mechanism by which the virus affects the gastrointestinal tract is still not so clear. The occurrence of several mechanisms has been hypothesized. One mechanism may involve the presence of ACE2 receptors in the gastrointestinal tract. Liang et al. found that ACE2 was highly expressed in the small intestine especially in proximal and distal enterocytes (207). In addition, Zhang et al. found that ACE2, TMPRSS2, and Furin, all critical for fusion of viral and the cellular membranes, were co-expressed in esophageal upper epithelial and gland cells and also in the enterocytes from ileum and colon, thus speculating exactly these organs as potential targets for SARS-CoV-2 (208). In addition, Guo et al. suggested that TMPRSS2 was highly expressed in almost all organs of the digestive tract including colon, stomach, small intestine, and esophagus (209). The co-expression of ACE2 and TMPRSS2 in the intestinal enterocytes may explain the disruption of intestinal absorption that leads to diarrhea. However, a second mechanism could involve a direct injury of the gastrointestinal system due to an inflammatory response (cytokine storm) (208). Absorptive enterocytes may be infected and destroyed by the virus, probably leading to malabsorption, disturbed intestinal secretion, and an activated enteric nervous system ensuing symptoms like diarrhea (210).

### Liver

On October 6, 2020 searching on PubMed “COVID-19 OR COVID-2019 OR severe acute respiratory syndrome coronavirus 2 OR severe acute respiratory syndrome coronavirus 2 OR 2019-nCoV OR SARS-CoV-2 OR 2019nCoV OR (Wuhan AND coronavirus) AND liver” we found 1,319 papers. Several data reported that approximately half of SARS-CoV-2 patients show liver biochemistry abnormalities, with increased levels of aminotransferases, gamma-glutamyl transferase, bilirubin, and alkaline phosphatase (116, 211–218). Median aspartate aminotransferase-dominant aminotransferase increase seems to indicate the disease severity and seems to be an index of hepatic injury (211). Concerning hepatic injury, Bloom et al. reported that about 1 in 5 patients developed grade 3 or 4 hepatocellular injury during hospitalization (212). In addition, it was reported that liver abnormalities seem to be more common in patients with severe disease upon presentation (212). In fact, a recent meta-analysis including 20 retrospective studies with 3,428 COVID-19 patients revealed that higher levels of alanine aminotransferase, aspartate aminotransferase and bilirubin were associated with a significant increase in the severity of COVID-19 infection (219). A recent meta-analysis also linked elevated admission levels of these markers to patient mortality (220). Other common factors linked with liver injury were decreased lymphocyte count, increase neutrophil count, and male gender (213). However, to date, the exact changes that lead to the altered liver biochemistries in SARS-CoV-2 patients remains unclear. Post-mortem liver biopsy showed the presence of a moderate microvascular steatosis and a mild lobular and portal activity (116). Another study suggested collateral liver damage from viral-induced cytotoxic T-cells (221). Additionally, since also abnormal coagulation markers have been reported in SARS-CoV-2 patients it is possible that the presence of microthrombi lead to an altered hepatic perfusion and consequent hepatocyte injury and aspartate aminotransferase increase (214, 215, 222). Whether these changes can be due to direct viral cytopathic effect, to cytokine release linked with SARS-CoV-2, to ischemia, to a preexisting condition, or to other causes, such as drug-induced liver injury, are currently unknown, also because studies on mechanisms of SARS-CoV-2 related liver dysfunction are limited. What we know currently is that ACE2 receptor are highly expressed in cholangiocytes (59.7%) and low expressed in hepatocytes (2.6%), thus some studies hypothesized a cholangiocytes mediating viral-associated injury (216). However, Zhou et al. showed that TMPRSS2 is highly expressed in hepatocytes (223). In fact, it was shown that alkaline phosphatase, an index of cholangiocytes injury, was the liver parameter less subject to significant alterations during SARS-CoV-2 infection while, aminotransferases and gamma-glutamyl transferase, indicators of hepatocyte injury, were the more common and almost always altered liver parameters in severe SARS-CoV-2 patients (116). In fact, autoptic analyses of liver tissue from SARS-CoV-2 patients do not demonstrate a cholangiocyte damage (116). As just described, liver injury in COVID-19 may be the direct insult to the liver or bile cells via receptors of ACE2 but it is further aided by hyper-inflammation, cytokine storm or bystander hepatitis and drug-induced damage. Another hypothesis is that since the SARS-CoV-2 RNA was also present in stool, it would be possible a transmission from the gut to liver by portal circulation (224). To date the exact mechanism of viral-associated liver injury needs further investigation.

### Gallbladder

On October 7, 2020 searching on PubMed “COVID-19 OR COVID-2019 OR severe acute respiratory syndrome coronavirus 2 OR severe acute respiratory syndrome coronavirus 2 OR 2019-nCoV OR SARS-CoV-2 OR 2019nCoV OR (Wuhan AND coronavirus) AND gallbladder” we found 21 papers. Despite few articles were found on gallbladder during SARS-CoV-2 infection, several information on its alteration during the new viremia were found in manuscripts on liver injury (225, 226). Gallbladder is a storage pouch for bile that is continually produced by liver, thus their functions are strictly related. Specific right upper quadrant ultrasounds on gallbladder of SARS-CoV-2 patients detected gallbladder sludge and distention in about 54% of patients, suggesting the presence of cholestasis (226, 227). Cholestasis in these patients seem to be not associated with age, gender, ICU admission, or gastrointestinal symptoms at presentation (226). The fatality rate seems to be higher among patients with cholestasis than those without cholestasis (228). As for liver, the gallbladder was found susceptible to the infection probably due to the high ratio of gallbladder epithelium cells expressing ACE2 (28). Also in this case the mechanism of viral-associated gallbladder alterations is unclear, although it is obvious that its alterations during SARS-CoV-2 infection are associated with liver injury.

### Pancreas

On October 7, 2020 searching on PubMed “COVID-19 OR COVID-2019 OR severe acute respiratory syndrome coronavirus 2 OR severe acute respiratory syndrome coronavirus 2 OR 2019-nCoV OR SARS-CoV-2 OR 2019nCoV OR (Wuhan AND coronavirus) AND pancreas” we found 77 papers. Currently, data on pancreas involvement in SARS-CoV-2 infection are scarce. However, several case reports showed pancreatic injury in COVID-19 patients and it was reported that about 1–2% of non-severe and 17% of severe patients with SARS-CoV-2 infection presented pancreatic injury (5, 229–231). Several of these patients also presented abnormal blood glucose, suggesting that the pancreatic injury might be due directly to cytopathic effect by local SARS-CoV-2 replication (5, 229–231). Additionally, pancreatic injury might be caused indirectly by systemic responses to respiratory failure or to the harmful immune response induced by SARS-CoV-2, which led also to the damage in multiple organs (5). Similar results were also found by Liu et al. that detected elevated levels of amylase and lipase associated to focal enlargement of the pancreas or dilatation of the pancreatic duct based on CT scans (232). Hadi et al. also described SARS-CoV-2 patients with severe acute pancreatitis, which itself may lead to multi-organ failure including adult respiratory distress and kidney failure as seen the patients examined in the study (231). Considering the proportion of SARS-CoV-2 patients with pancreatic injury and theexpression of ACE2 and TMPRSS2 in the pancreas (particularly in pancreatic islet cells), researcher and clinicians should pay attention to the possibility of damage caused by SARS-CoV-2.

### Brain

On October 7, 2020 searching on PubMed “COVID-19 OR COVID-2019 OR severe acute respiratory syndrome coronavirus 2 OR severe acute respiratory syndrome coronavirus 2 OR 2019-nCoV OR SARS-CoV-2 OR 2019nCoV OR (Wuhan AND coronavirus) AND brain” we found 1,293 papers and most of them showed that SARS-CoV-2 invades the CNS, developing neurological impairments such as stroke, epilepsy, anosmia and hypogeusia, seizures, and encephalitis (1, 66, 233–236). Specifically, a retrospective analysis by Mao et al. (237) underlined that about 40% of SARS-CoV-2 patients developed headache, disturbed consciousness, and other brain dysfunction symptoms (1), and an autopsy study reported the presence of edema in brain tissue of SARS-CoV-2 patients (66). Several case-series and two retrospective studies also reported critical stroke conditions related to COVID-19 (238, 239). In this context, Beyrouti et al. examining a small cohort of COVID-19 patients, also underlined that ischemic stroke (confirmed by reverse-transcriptase PCR) linked to severe SARS-CoV-2 patients occurs in the context of a systemic highly prothrombotic state, as shown by large vessel occlusion and elevated D-dimer levels (240), conditions that can make patients more prone to acute cerebrovascular events. Moriguchi et al. reported the first case of meningitis related to COVID-19 underling that SARS-CoV-2 RNA was not present in nasopharyngeal swab, but it was detected in cerebrospinal fluid sample (241). Numerous cases of encephalitis/encephalopathy associated with SARS-CoV-2 infection were also described and were confirmed by post-mortem analyses, where acute disseminated encephalomyelitis and neocortical micro-infarcts were detected (242–244). Neurologic complications associated to COVID-19 patients are not limited to the CNS. In fact, several authors also reported a correlation between SARS-CoV-2 and Guillan-Barrè syndrome, an acute/sub-acute immune-mediated polyradiculoneuropathy with diverse degrees of limbs or cranial-nerves weakness, lack of deep tendon reflexes, sensory, and dysautonomic symptoms cause by peripheral nerves and roots demyelination and/or axonal injure (245–248). Other studies also described Miller Fisher syndrome as another neurologic complication of SARS-CoV-2 infection (249–251). These neurological manifestations in the brain of SARS-CoV-2 infected patients were confirmed and recognized by CT scan images and magnetic resonance imaging (MRI) scan, where presence of necrotizing hemorrhagic encephalopathy, brain thrombosis and acute infarction, eptomeningeal enhancement, perfusion abnormalities, and cerebral ischemic stroke, demyelinating lesions, right temporal lobe edema, and brainstem inflammation, were recognized (252–255). In addition, the presence of SARS-CoV2 was identified in frontal lobe tissue by using transmission electron microscopy (237) and by genome sequencing in cerebrospinal fluid of SARS-CoV-2 patients, supporting that this new pneumonia virus can cause nervous system damage (241). In addition to the above described neurological manifestations, several SARS-CoV-2 infected patients showed delirium and/or mental status changes. These symptoms may be a manifestation of direct CNS invasion, induction of CNS inflammatory mediators but may be also a secondary effect of other organ system failure, an effect of sedative strategies, a prolonged mechanical ventilation time, or environmental factors, including social isolation (256). Despite these data clearly highlighted the involvement of the brain in SARS-CoV-2 infection, the exact mechanism for virus neurotoxicity is not yet straightforward, since this depend on the brain entry route of the virus, which, to date, has not been fully elucidated (257). The pathway of the virus into the brain could be primarily linked to the route of transmission and distribution of intracellular receptors of SARS-CoV-2. Mao et al. hypothesized that SARS-CoV-2 virus may interact with ACE2 in the capillary endothelium and caused blood–brain-barrier destruction, thus promoting the entry of the virus into CNS (237) and next causing neuroinfection. In fact, it was found that ACE2 and TMPRSS2 were expressed in the oligodendrocyte precursor cells and the astrocytes of the substantia *nigra* and cortex (82). COVID-19 can potentially damage the capillary endothelium within the brain and contribute to elevated blood pressure. The risk of SARS-CoV-2 cerebral hemorrhage through an ACE2 receptor can result in abnormally high blood pressure and increase cerebral hemorrhage. However, although ACE2 and TMPRSS2 are present in the nervous system, additional pathways were also hypothesized for the entry of SARS-CoV-2 into the nervous system, including the direct intranasal entry to the brain *via* olfactory nerves, the indirect entry to the brain go through the blood-brain barrier via hematogenous or lymphatic spread, the hypoxic injury, and finally the immune-related injury (7, 258). It is known, that coronaviruses can enter to the nervous system straight through the olfactory nerve, potentially causing loss of smell and taste, and enter the nervous system through blood circulation and neuronal pathways. In addition, coronaviruses, including SARS-CoV-2, trigger harmful effects in the lung tissue leading to several lung lesions and consequent hypoxia, that can be responsible of the brain disease progression. These data highlighted that awareness, management and timely analysis of infection-related neurological complications of SARS-CoV-2 patients are key to improve the prognosis of severe ill patients.

### Skin

On October 7, 2020 searching on PubMed “COVID-19 OR COVID-2019 OR severe acute respiratory syndrome coronavirus 2 OR severe acute respiratory syndrome coronavirus 2 OR 2019-nCoV OR SARS-CoV-2 OR 2019nCoV OR (Wuhan AND coronavirus) AND (skin OR cutaneous manifestation) we found 771 reports. Skin manifestations due to SARS-CoV-2 infection are of different types and currently reported in numerous case reports, case series, and literature reviews (259–264). The first case study on skin manifestations was published by Recalcati et al. and included 88 patients that showed widespread urticaria, erythematous rush and chickenpox-like vesicles (265). Subsequently, other authors described urticarial rash petechial also in association with decrease platelet count and sometimes also with eosinophilia (265–270). Zhang et al. evaluating 140 patients with SARS-CoV-2 infection, stated that urticaria were self-reported by 1.4% of patients (268). Despite, the majority of studies reported that urticarial skin manifestations were not correlated with SARS-CoV-2 severity (265, 268), a prospective cohort study reported that the presence of urticaria and maculopapular skin lesions were associated with higher morbidity and higher mortality rate (2%) (271). In addition to urticarial skin manifestations, Manalo et al. also described a transient livedo reticularis as potential skin manifestation linked to SARS-CoV-2 (272). Other described skin manifestations are related to acral ischemia often related to an hypercoagulation status of SARS-CoV-2 patients, that have a negative prognostic implication in virus evolution (273–275). These manifestations could be caused by direct injury of vascular endothelium by SARS-CoV-2, which could lead to DIC, antiphospholipid syndrome, and vasculitis mimics. Case series showed purpuric skin involvement in severe SARS-CoV-2 patients, in detail retiform purpura on the buttocks, dusky purpuric patches on the palms and soles, and livedo reticularis on the chest and limbs were detected (261, 273, 276). Tissue biopsies from skin and lung detected thrombogenic vasculopathy and deposits of C5b-9 and C4d complement proteins (273). This was in line with widespread activation of both alternative and lectin pathways of complement, suggesting that severe SARS-CoV-2 patients can suffer thrombotic microvascular injuries that can involve not only the lungs but also the skin, and probably other organs (273). Skin manifestations were found also in pediatric patient where the skin lesions commonly happen in asymptomatic or mildly symptomatic children and adolescents (277–279). Skin biopsy of acral perniosis lesion in SARS-CoV-2 pediatric patients revealed a superficial and deep lymphocytic infiltrate, where vacuolar change and purpura were also present (280, 281). Hemorrhagic parakeratosis in the stratum corneum were also detected and as well as dermal infiltrate strongly perivascular and perieccrine and lymphocytic vasculitis in the thin muscular walls of small vessels (4, 205). Similar results were also found in skin biopsies from SARS-CoV-2 adult patients that showed a lymphocytic perivascular and perieccrine infiltrate (282, 283). To date, there are still not enough studies to define which are the skin manifestations of SARS-CoV-2 infection, and why they occur. As reported by Recalcati et al. these dermatological manifestations “are similar to cutaneous involvement occurring during common viral infections” (265). Several hypotheses could be formulated from integration of the clinical observations and data from literature, but to date whether these skin manifestations were neurogenic, microthrombotic, or immune complex mediated is unclear. However, examine the tissue samples to understand if SARS-CoV-2 can be detected in the skin itself could be of key importance also considering that ACE2 is present in basal epidermal layers and in sebaceous gland cells of the skin (58). In addition, a recent study detected that ACE2 and TMPRSS2 were co-expressed at the epithelial sites of the skin, highlighting the potential roles of these molecules in SARS-CoV-2 (284). However, so far it is not known if skin manifestations (non-pruritic, erythematous rashes, urticaria, or varicella-like lesions) in COVID-19 patients are a place of viral replication or just a local reaction to systemic infection.

### Male and Female Reproductive System and Pregnancy

On October 7, 2020 searching on PubMed “COVID-19 OR COVID-2019 OR severe acute respiratory syndrome coronavirus 2 OR severe acute respiratory syndrome coronavirus 2 OR 2019-nCoV OR SARS-CoV-2 OR 2019nCoV OR (Wuhan AND coronavirus) AND (reproductive system OR ovaries OR testis OR pregnancy)” we found 1,301 reports. Most of these reports described high levels of ACE2 expression in the testes, spermatids, ovaries, fallopian tubes, placenta, and uterus, thus highlighting a potential high risk of SARS-CoV-2 infection in the human reproductive system (61, 285–288). However, data on the presence of SARS-CoV-2 in male reproductive system are conflicting. A study carried out by Li et al. revealed that SARS-CoV-2 was found in the testes of infected cases (289). A post-mortem study on 91 COVID-19 victims also showed varying degrees of spermatogenic cell reduction and damage, and presence of SARS-COV-2 RNA and virus particles in the testes (290). Conversely, some clinical studies did not detect SARS-CoV-2 in semen or testicular biopsy of COVID-19 patients (291–293). Thus, it is possible to speculate that SARS-CoV-2 gains access to the male reproductive system in some but not in every COVID-19 patient. However, since SARS-CoV-2 can lead to systematic effect it can have also other consequences on the reproductive system. Several studies reported testicular discomfort and devastation to the testicular parenchyma in COVID-19 patients even when the testes were SARS-CoV-2 negative (291, 293).

As known, the reproductive health issues may not be restricted to men, but woman may also have consequences. What seems to be quite clear is the distribution and function of ACE2 in the female reproductive system. Jing et al. clearly reported the ACE2 expression in the ovary, uterus, vagina, and placenta (60). Moreover, since Ang II, ACE2, and Ang-(1–7) regulate follicle development and ovulation, modulate luteal angiogenesis, and degeneration, and influenced the regular changes in endometrial tissue and embryo development SARS-CoV-2 infection may disturb the female reproductive functions, resulting in infertility, menstrual disorder and fetal distress (60). Although these data suggested that there are potential routes for SARS-CoV-2 to compromise female fertility, currently no studies on damage to female COVID-19 patients' reproductive system were reported. For SARS-CoV-2 role in female reproductive system, the latest evidences were mainly focused on pregnant women. The simultaneous expression of ACE2 and TMPRSS2 seems to lack at the cellular level of maternal- fetal interface. Despite the clinical manifestation in COVID-19 pregnant women seems to remain the same as in non-pregnant patients, several studies suggest that pregnant women infected with COVID-19 may be at risk for preterm delivery (294–296). Recent papers also reported cases of pre-eclampsia and manifested gestational hypertension in COVID-19 pregnant women (297–299). An analysis of the WAPM study on COVID-19 reported that early gestational age at infection, maternal ventilatory supports and low birthweight are the main determinants of adverse perinatal outcomes in fetuses with maternal COVID-19 infection (300). However, significant neonatal respiratory diseases appear to be rare in presence of SARS-CoV-2 positivity (301). In this context, the key question is whether SARS-CoV-2 can be transmitted to fetuses from a woman infected with COVID-19. The evidence of infection in infants in the time immediately following birth (since few hours to a couple of days) suggests the possibility of maternal fetal transmission, via intrauterine vertical infection or mediated by breastfeeding. In this latter case, evidence of the presence of Sars- Cov2 in maternal milk is still controversial, and in the close contact between mother and child in these phases could lie the true way of transmission. Instead, despite primary reports from China suggested that vertical transmission was unlikely, several case series revealed the possibility of vertical transmission from positive SARS-CoV-2 woman (302, 303). Two conditions are mandatory for transplacental transmission to be possible: (1) SARS-CoV-2 must reach the placenta and (2) ACE2 must be present in the placenta. Regarding the first condition, several papers supported the presence of SARS-CoV-2 in placental tissue. In particular, histopathological signs of placenta alteration have been observed in pregnant women affected by Sars-CoV-2, with evidence of inflammatory state and alteration in vascular supply. (304–308). Regarding the second condition controversial results are still present (309, 310). However, a recent study indicated that trophoblastic cells, which are in direct contact with the maternal blood in the intervillous space, showed a strong expression of ACE2 throughout pregnancy, supporting that SARS-CoV2 is able to infect the placenta *via* a receptor-mediated mechanism (311). A further study investigated the potential transmission routes in the first trimester, and they found expression of ACE2 and co-expression of TMPRSS2 in the trophoblast, blastocyst, and hypoblast (312). However, other proteases such as Furin, trypsin and cathepsins B and L could be also implicated (16, 313, 314). Thus, despite the lack of clinical evidence, SARS-CoV-2 infection may carry a potential risk of reproductive system.

### Thyroid

On October 7 2020 by searching on PubMed “COVID-19 OR COVID-2019 OR severe acute respiratory syndrome coronavirus 2 OR severe acute respiratory syndrome coronavirus 2 OR 2019-nCoV OR SARS-CoV-2 OR 2019nCoV OR (Wuhan AND coronavirus) AND thyroid” we found 112 papers. Data on direct thyroid involvement in SARS-CoV-2 infection arescarce and most of the reports are focused on identifying a possible association between hypothyroidism and outcomes related to COVID-19. A consensus statement regarding issues specific to thyroid dysfunction during SARS-CoV-2 pandemic was issued by the British Thyroid Association and the Society for Endocrinology (315). The consensus suggested to patients with hypothyroidism or hyperthyroidism to continue their medications, however, it underlined that patients on anti-thyroid drugs are at a risk of agranulocytosis, symptoms that often overlap with those of SARS-CoV-2 (315). However, recently, van Gerwen et al. evaluated 3,703 COVID-19 patients of which 251 patients (6.8%) had pre-existing hypothyroidism and received thyroid hormone therapy (316). They found that hypothyroidism was not associated with increased risk of hospitalization, mechanical ventilation, and death (316). A direct thyroid involvement associated with COVID-19 was highlighted by Campos-Barrera et al. that identified a subacute thyroiditis associated with a very mild presentation of COVID-19 in a healthy 37-year-old female (317). Subacute thyroiditis was not the only thyroid condition associated with COVID-19. In fact, cases of thyroxine thyrotoxicosis have been also described (318). Several case reports and a case series were focused on the prevalence of subacute thyroiditis and thyroxine thyrotoxicosis in patients with severe presentation of COVID-19 from ICU (319–324). More recently in a retrospective study on 50 COVID-19 patients it was found a decrease in total T3 and TSH concentrations in 56% of patients (325–327). The decrease in T3 concentration resulted more pronounced in patients with the severe SARS-CoV-2 (325). Despite the few data related to the thyroid involvement during SARS-CoV-2 infection, it is important to emphasize that, as previously reported ACE2 expression levels were high in thyroid and its expression were positively and negatively associated with immune signatures in males and females (328). Additionally, TMPRSS2 was also expressed in thyroid (82). Therefore, surely further studies would be important to understand a potential involvement of the thyroid in SARS-CoV-2 infection.

## Discussion

Since it has been demonstrated that the novel SARS-CoV-2, which affected a very high number of people all over the world, entry into the cell exploiting ACE2, more and more research and studies are focusing their attention on ACE2 role, function, and distribution and on its interaction with specific proteases that assist SARS-CoV-2 infection. In fact, it is known that following the entry of the virus into the human cell through the binding with ACE2, the S protein is cleaved by TMPRSS2, with the help of Furin which facilitates the entry of the virus into the cell after binding. However, theoretically, also other human's proteases (cathepsin L and B, elastase, trypsin and factor X) could be involved in this complex process and numerous studies are currently ongoing.

Our overview highlighted that ACE2 receptors, being ubiquitous, and extensively expressed in numerous human tissues and organs, such as in the heart, vessels, gut, lung, kidney, testis, and brain and many other, may play a key role in the involvement and subsequent impairments of various organs during the SARS-CoV-2 infection. ACE2 is typically bound to cell membranes and poorly present in the soluble form in circulation. In addition to its negative role in SARS-CoV-2 infection, and in other virus, membrane-bound and soluble ACE2 also perform beneficial biological functions, the main represented by the degradation of angiotensin II to angiotensin 1–7. Thus, ACE2 receptors cut down some harmful effects consequential to the bind of angiotensin II to AT1 receptors, which comprise vasoconstriction, increase inflammation, and thrombosis (329). However, the entry of SARS-CoV-2 in the cells by membrane fusion down-regulates ACE2 receptors, thus SARS-CoV-2 seems to entry into the cell with the membrane receptor, which is functionally detached from the membrane external site. This phenomenon can cause the detrimental effects in SARS-CoV-2 infection. It is important to underline that several other factors, such as genetics, demographic, lifestyle, co-morbidities and drugs usage could have a potential impact on ACE2 expression and activity. In fact, it was extensively reported that SARS-CoV-2 patients present several features associated with infection and severity of the disease, such as older age, hypertension, diabetes and cardiovascular disease, that share a different degree of ACE2 deficiency and that can produce bias in the evaluation of the effective damages caused by the virus (1). However, despite during SARS-CoV-2 infection ACE2-expressing organs may become direct targets, leading to critical pathological manifestations and subsequent multiple organ failure or even death, the exact mechanism and effective action through which ACE2 act on these organs is still heavily debated. Another point at the center of the clinical and scientific debate is represented by the potentially beneficial effect (or not) of soluble form of ACE2, the form that lacks the membrane anchor and circulates in small amounts in the blood. A paper by Battle et al. hypothesizes that the soluble form of ACE2 might behave like a competitive interceptor of SARS-CoV avoiding the binding of the virus to the surface-bound, full-length ACE2, the form that contains a structural transmembrane domain, which anchors its extracellular domain to the plasma membrane (330). This evidence is in line also with in *vitro* studies (331, 332). A preclinical model of Vero-E6 cells, infected with SARS-CoV-2, isolated from a nasopharyngeal sample of COVID-19 patient, demonstrated the efficacy of human recombinant soluble ACE2 (hrsACE2) in inhibiting viral replication in a dose-dependent manner. Such activity was also confirmed in human capillary organoids cultures and in kidney organoids cultures generated from human embryonic stem cells (331). In addition, the soluble ACE2 form seems to be also involved in blocking SARS-CoV-2 replication and in immune response against the virus, in concert with Fc portion of immunoglobulin (331). The administration of rhACE2 also seems to induce a reduction of IL-6 levels in severe COVID-19 patients (333). The increased production of IL-6 and other inflammatory cytokines (IL-1β, IL-2, IL-7, IL-8, IL-10, granulocyte-colony stimulating factor, granulocyte macrophage-colony stimulating factor, interferon-inducible protein-10, monocyte chemotactic protein 1, macrophage inflammation protein-1α, IFN-γ, and TNF-α,2,3,12,15) together with the presence of lymphopenia, lymphocyte activation and dysfunction, abnormalities of granulocytes and monocytes, increased production of immunoglobulin G (IgG) and total antibodies in COVID-19 patients points out how the SARS-CoV-2 is able to disrupt also the normal immune responses, leading to an impaired immune system (334–339). Lymphopenia is a key feature of patients with severe COVID-19 (334). A marked reduction in the number of CD4+ T, CD8+ T, NK, and B cell was detected in these patients (335). In addition, a high expression of CD69, CD38, and CD44 on CD4+ and CD8+ T cells was seen (336). Virus-specific T cells from severe COVID-19 patients also highlight a central memory phenotype with high levels of interferon (IFN)-γ, tumor necrosis factor (TNF)-α, and IL-2. Nevertheless, lymphocytes have an exhaustion phenotype with programmed cell death protein-1 (PD1), T cell immunoglobulin domain and mucin domain-3 (TIM3), and killer cell lectin-like receptor subfamily C member 1 (NKG2A) upregulation (337). Unlike eosinophils, basophils, and monocytes percentage that were reduced in severe COVID-19 patients the level of neutrophils resulted increased (338). Thus, the damage and inefficiency of the immune system caused by lymphopenia, T cell exhaustion and cytokine release syndrome, and organ specific ACE2 expressing cells (endothelial, alveolus in lungs, proximal tubule, and glomerulus in kidneys, pericytes in heart, ect) could potentially lead to complications like acute respiratory disease syndrome and multi-organ failure. These complications not only can lead to a poorer outcome to the SARS-CoV-2 infection but can also lead to permanent alterations that can persist long after viremia (*long-term COVID-19*), such as pulmonary fibrosis, neurodegenerative deseases, cardiovascular and kidney diseases (340, 341). Highlighting the pathological basis and mechanisms of COVID-19 and all the functions and activities of ACE2 during the virus would be essential for our understanding of the pathophysiology of the disease. A great help to our understanding could come from pathological studies of larger series of autopsy findings. Furthermore, the development of advanced and alternative preclinical models could help to discover more about the SARS-CoV-2 infection process itself, to analyze specific aspects of ACE2 in relation to SARS-CoV-2 pathophysiology and, most importantly, to learn the disease progression pattern observed in humans. In addition, more exhaustive and systematic studies on the physiological localization and activity of ACE2 might help in the comprehension of the mechanisms underlying the infection. In this regard, attention should be paid to the investigation of the cells of the immune system, in consideration of preliminary scientific evidence on the identification of ACE2 in immune cells residing in the tissues. This could open further scenarios on both the virus spreading mechanisms and tissues damage.

We believe that devote scientific efforts for the clinical management of SARS-CoV-2 patients, also considering a personalized strategy aimed to provide individually tailored treatment for each patient, are currently mandatory. As showed in this report this aspect should also considered specific patient differences in the mutual interactions ACE2-SARS-CoV-2 with their consequences for the disease pathophysiology. Another interesting aspect that could be explored in patients who have overcome the disease is the possible onset or persistence of the alterations above described in the organs and systems and the evaluation of whether they are transient or permanent (*long-term COVID-19*), to assess the extent of ACE2 activity impairment due to SARS-CoV-2 infection.

## Author Contributions

FS, MF, and ML designed the manuscript. FS and MM collected and analyzed literature, wrote the manuscript, edited, and prepared manuscript for submission. ML and MF revised the manuscript. All authors read and approved the final manuscript.

## Conflict of Interest

The authors declare that the research was conducted in the absence of any commercial or financial relationships that could be construed as a potential conflict of interest.

## Footnotes

^1^https://news.joins.com/article/23738003?cloc=joongang-mhomegroup6 (accessed July 15, 2020)
